# Quantifying Auditory Temporal Stability in a Large Database of Recorded Music

**DOI:** 10.1371/journal.pone.0110452

**Published:** 2014-12-03

**Authors:** Robert J. Ellis, Zhiyan Duan, Ye Wang

**Affiliations:** School of Computing, National University of Singapore, Singapore, Singapore; University of Texas Health Science Center at San Antonio, Research Imaging Institute, United States of America

## Abstract

“Moving to the beat” is both one of the most basic and one of the most profound means by which humans (and a few other species) interact with music. Computer algorithms that detect the precise temporal location of beats (i.e., pulses of musical “energy”) in recorded music have important practical applications, such as the creation of playlists with a particular tempo for rehabilitation (e.g., rhythmic gait training), exercise (e.g., jogging), or entertainment (e.g., continuous dance mixes). Although several such algorithms return simple point estimates of an audio file’s temporal structure (e.g., “average tempo”, “time signature”), none has sought to quantify the temporal *stability* of a series of detected beats. Such a method-a “Balanced Evaluation of Auditory Temporal Stability” (BEATS)–is proposed here, and is illustrated using the Million Song Dataset (a collection of audio features and music metadata for nearly one million audio files). A publically accessible web interface is also presented, which combines the thresholdable statistics of BEATS with queryable metadata terms, fostering potential avenues of research and facilitating the creation of highly personalized music playlists for clinical or recreational applications.

## Introduction

With the proliferation of back-end warehouses of music metadata (e.g., AllMusic, Gracenote, Last.fm, MusicBrainz, The Echo Nest [1]), front-end online music stores (e.g., Amazon MP3, Google Play Music, iTunes, 7digital, Xbox Music [2]), and streaming music services (e.g., Deezer, MySpace Music, Napster, Rdio, Rhapsody, Spotify [3]) comes heretofore unparalleled opportunities to change the way music can be personalized for and delivered to target users with varying needs.

One need, shared by both rehabilitation professionals and exercise enthusiasts, is the ability to create music playlists which facilitate the synchronization of complex motor actions (e.g., walking) with an auditory beat. Auditory-motor synchronization has been deemed a human cultural universal [4] and a “diagnostic trait of our species” [5]. Even infants show perceptual sensitivity to [6] and coordinated motor engagement with [7] musical rhythms. The phenomenon of auditory entrainment (the dynamic altering of an “internal” periodic process or action generated by an organism in the presence of a periodic acoustic stimulus) remains an active topic for the field of music cognition [8]–[14].

Auditory-motor synchronization has received particular interest in the context of preventive and rehabilitative physical exercise, with a number of advantages for participants (for recent summaries, see [15]–[17]): cognitively, by focusing attention (cf. [18]–[20]); motivationally, by increasing arousal (cf. [21], [22]), endurance during a session (e.g., [23], [24]), and adherence across sessions (e.g., [25], [26]); and socially, by enabling multiple individuals to participate and interact in a coordinated manner, as in partnered or group dancing (e.g., [27], [28]).

A particularly successful application of auditory-motor synchronization paradigms has been for patients with Parkinson’s disease (PD), where it is referred to as “Rhythmic Auditory Stimulation” or “Rhythmic Auditory Cueing” (RAC). Although the facilitative effects of an external auditory cue on parkinsonian gait had been noted anecdotally since the 1940 s (e.g., [30], [31]), experimental work in the 1990 s (e.g., [32], [33]) and subsequent multi-week clinical trials (e.g., [34], [35]), systematic reviews [36], [37], meta-analyses [38], [39], and evidence-based “best practice” treatment recommendations [40] have all pointed towards RAC as a reliable and effective means of improving several features of gait: increasing cadence, stride length, and velocity (as reviewed in [38], [39]); and decreasing gait *variability* (i.e., moment-to-moment fluctuations in step timing or step length; for comprehensive reviews, see [41]–[43]). A reduction in gait variability is of particular importance, as it is linked both retrospectively [44] and prospectively [45] with a reduced likelihood of falling, a costly event both financially (e.g., [46]) and psychologically (e.g., [47]). Although less well-explored, RAC-mediated improvements in gait have also been noted for other neurological conditions, including Huntington’s disease [48], [49], stroke [50], [51], spinal cord injury [52], and traumatic brain injury [53]. (For a systematic review of this evidence, see [54].).

### 1. Physical Isochrony versus Perceptual Stability

A basic requirement for the music used in auditory−motor rehabilitation paradigms is it possesses a stable *tempo* (i.e., the rate at which beats or pulses are perceived to occur), thereby facilitating motor synchronization to the beat. This requirement is typically satisfied through the use of a digital metronome, either in isolation or superimposed on top of computer-generated music (e.g., [51]), ensuring a precisely isochronous inter-beat interval (IBeI). However, a slightly more relaxed requirement could be proposed: that the sequence of IBeIs in the music stimulus need not be physically *isochronous*, but rather, be perceptually *stable*.

Systematic investigations of just-noticeable differences (JNDs) or other perceptual discrimination thresholds of anisochrony in auditory temporal sequences date back several decades (for reviews, see [13], [14], [55]–[57]). A wide range of stimuli has been explored:

(1) isolated time intervals (e.g., [58], [59]); (2) a single temporal perturbation within an isochronous (e.g., [55], [56], [60], [61]) or anisochronous (e.g., [20], [62]) context; (3) a single tempo change between a pair of monotonic isochronous sequences (e.g., [62]–[65]) or excerpts of computer-performed, quantized music [66]; (4) a pair of sequences, one isochronous and the other with Gaussian temporal “jitter” [67]; (4) continuously cosine-modulated temporal intervals [68]; and (5) continuously accelerating or decelerating sequences (e.g., [69]–[71]). In general, JNDs for anisochrony decrease as the number of repetitions of a fixed temporal interval increases, and are higher overall within sequences in which temporal instability is present.

Although these conditions are well-controlled experimentally, they do not necessarily generalize to *performed* music. That is, absent from a digitally produced rhythm track, it would be expected that IBeIs in performed music exhibit some degree of “natural” variability in tempo (or, perhaps less pejoratively, “flexibility in tempo”). However, an important question that follows from this assumption–namely, “How much physical variability in an IBeI sequence results in the perceptual instability of tempo?”–has not been clearly asked, or answered. By contrast, studies seeking to quantify listeners’ perceptions of tonal stability (e.g., [72], [73]), or overall “musical stability” (e.g., [74]) are more frequent.

### 2. Beat Tracking and Tempo Extraction Algorithms

Accurately estimating the tempo of recorded music is an important topic within the field of music information retrieval (e.g., [75]–[77]), and numerous algorithms have been developed to accomplish this (for summaries, see [78]–[81]). Two broad categories of algorithms can be defined. *Beat tracking* algorithms return a time series of detected IBeIs along with a point estimate of “average” tempo in beats per minute (bpm). *Tempo extraction* algorithms return only the latter.

An important goal for beat tracking algorithms is to identify the temporal locations of each beat accurately (i.e., with respect to listeners’ “ground truth” perceptions) in the face of changes, drifts, fluctuations, or expressive variations in tempo within an audio file. The ability of a beat tracking algorithm to accurately *identify* the precise location of each beat in the face of a fluctuating temporal surface, however, is independent from its ability to meaningfully *quantify* how much temporal instability is actually present in the series of detected beats. Similarly, the ability of a tempo extraction algorithm to provide a point estimate (e.g., “tempo = 90 bpm”) that agrees with human perception (e.g., the average inter-tap interval when listeners were instructed to tap to the beat) reveals nothing about whether that estimate is stable across the entire audio file; and if not, over what time indices of the file that estimate *is* stable. (The accuracy of any point estimate is of course dependent upon the manner in which it was computed, as will be illustrated in Section 4 of the Methods.).

To our knowledge, no current software algorithm, front-end interface, or back-end metadata service provider has offered any statistic explicitly designed to quantify the amount of *beat-to-beat temporal instability* within an IBeI series.

To address this issue, we expand upon our previous conference paper [82] and present a novel analysis tool: a “Balanced Evaluation of Auditory Temporal Stability” (BEATS). BEATS itself does not perform beat tracking, but instead takes beat and barline (i.e., downbeat) onsets estimated by an independent beat tracking algorithm as input. For its initial release, BEATS has been optimized to the data structure of the “Million Song Dataset” [83] (MSD; http://labrosa.ee.columbia.edu/millionsong/), a publicly available collection of computed acoustic features (e.g., individual beat and barline onsets; average tempo; estimated time signature) and music metadata (e.g., artist, album, and genre information) associated with nearly one million audio files processed using the proprietary “Analyze” algorithm [84] developed by The Echo Nest (www.echonest.com). Compatibility with this data structure has scalable advantages, as the full Echo Nest library contains over 35 million analyzed audio files.

For each analyzed audio file, BEATS computes nine *Summary Statistics* that quantify some characteristic of the inter-beat or inter-bar interval data. These statistics can in turn serve as input to search engines for which tempo is a key query feature (e.g., [75], [85]–[87]).

By providing a more comprehensive quantitative analysis of both tempo *and* tempo stability, and incorporating those statistics as filterable features within an online resource (“iBEATS”, described in Section 3 of the Results), BEATS becomes a further step towards a solution that provides users with access to music that has been tailored to their (or their patients’) recreation or rehabilitation needs.

## Methods

### 1. Platform

BEATS is implemented in Matlab (version ≧7.8), supplemented by a few publicly available functions associated with the Million Song Dataset [88] and Matlab Central (http://www.mathworks.com/matlabcentral).

### 2. Raw Data

For each metadata file, BEATS pulls four Echo Nest fields: beats_start and bars_start (the estimated onsets of successive beats and barlines, respectively); and tempo and time_signature (point estimates directly provided by Echo Nest). Next, beats_start and bars_start are transformed into an inter-beat interval inter-bar interval series, respectively, by taking the first-order difference of each timestamp vector.

### 3. Initialization Thresholds

BEATS requires the user to specify three Initialization Thresholds:

1. “Local Stability Threshold”, θ_Local_: a percentage value (default = 5.0%) used to define the upper bound of what is deemed temporally stable at the level of individual and successive IBeIs (detailed below).
2. “Run Duration Threshold”, θ_Run_: the minimum duration (default = 10 s) of a set of adjacent IBeIs (i.e., a “Run”) that all fall below θ_Local_.
3. “Gap Duration Threshold”, θ_Gap_: the maximum duration (default = 2.5 s) between the last element of Run*_j_* and the first element of Run*_j_*_+1_.

### 4. Internal Calculations

**Figure 1:**
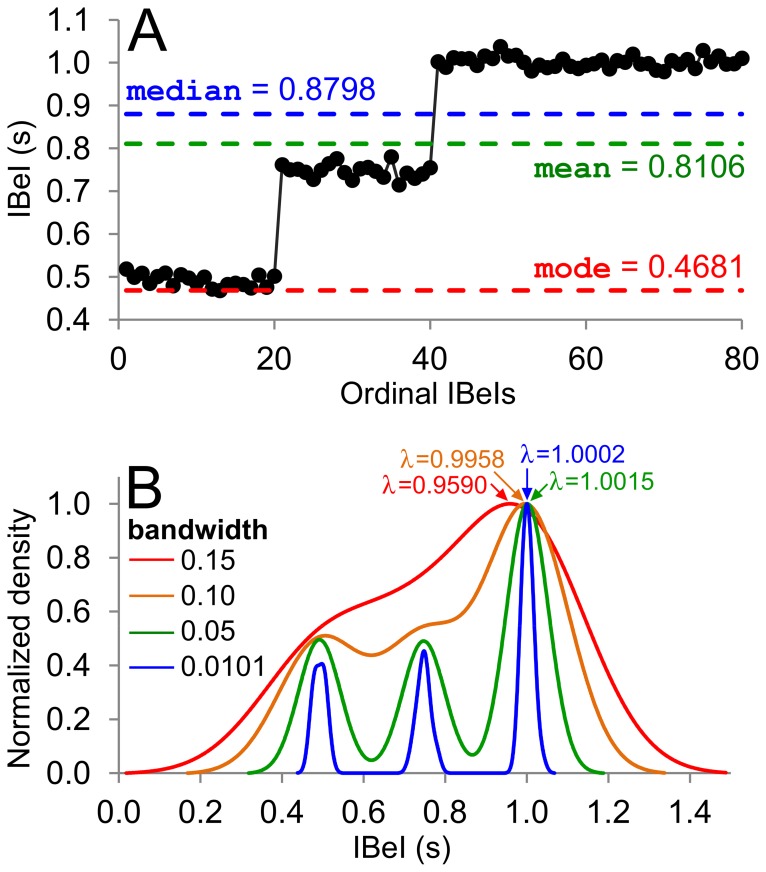
Illustrating different central tendency statistics. (A) A hypothetical IBeI series comprised of three distinct tempo sections: 20 IBeIs with a mean of 0.5 s (i.e., 120 bpm), followed by 20 IBeIs with a mean of 0.75 s (80 bpm), followed by 40 IBeIs with a mean of 1.00 s (60 bpm). The mean, median, and mode of the data fail to provide an adequate measure of central tendency. (B) Kernel density estimation (KDE) of the distribution of IBeI values in Figure 1A, using various bandwidth values. The most accurate measure of central tendency was obtained using adaptive Gaussian KDE [90], [91].

The first statistic calculated by BEATS is an estimate of an IBeI series’ central tendency, or *location*, λ. Common measures of λ include the mean, median, and mode. However, obtaining an optimal value for λ can be more complicated than simply taking the mean, median, or mode of a series. Consider the hypothetical 80-element IBeI series **S** shown in **Figure 1A**, which exhibits two tempo changes (at the 21st and 41st elements). Visual inspection of the Matlab-derived mean, median, and mode reveals that all are clearly inadequate measures of the “true” central tendency of **S** (i.e., ≈ 1.0).

One widely used method of obtaining a more accurate value for the central tendency of a dataset (specifically, the mode) has been the use of kernel density estimation (KDE) techniques, first proposed in the 1960 s [89] **Figure 1B** plots the estimated probability density of the distribution of values in **S**, using various values for the kernel *bandwidth* (i.e., the smoothing parameter). The mode of **S** is defined simply: the *x*-axis value at which the highest probability density (*y*-axis) occurs. As can be appreciated from Figure 1B, the bandwidth plays a strong role in the resultant mode: too narrow, and the mode will default to its most frequent value; too wide, and the density estimate will “smooth over” distinct features (in this case, time-varying features) within the data set, such as the presence of multiple modes.

To circumvent this problem, and thus provide a more “representative” value for λ, BEATS makes use of a recent implementation of adaptive (variable-bandwidth) Gaussian KDE [90], [91], which optimizes the bandwidth so as to return a valid density estimate even in the presence of multiple modes. Using this approach (shown as the blue density estimate in Figure 1B), λ is calculated as 1.0002: a far more representative value.

Having determined λ, the longest “Stable Segment” within the IBeI series is then identified. The first step in this process is to identify the locations of “stable” IBeIs, where stability is operationalized in two ways: stability of each IBeI relative to λ, and stability between successive IBeIs. The first type of stability is quantified via a “percentage deviation from λ” (PDL) transformation:
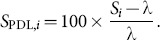
(1)


The second type of stability is quantified via a “successive percentage change” (SPC) transformation between IBeIs *i* and *i*+1:
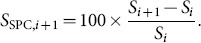
(2)


(Both **S**_PDL_ and **S**_SPC_ are expressed as relative percentages so as to facilitate comparisons across IBeI sequences in different tempo ranges.) These two equations are used in sequence to identify the location of temporally stable IBeIs. First, an *initial* determination of stability is made for each IBeI:
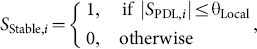
(3)where “1” indicates a stable IBeI relative to λ. Next, for all pairs of elements {*i*, *i*+1} for which **S**_Stable,*i*_ has a value of {1, 1}, **S**_Stable,*i*+1_ is then *revised*:



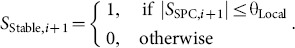
(4)A “Run” (i.e., a string of 1 s) within **S**_Stable_ thus indicates both temporal stability relative to λ as well as between successive IBeIs; a “Gap” (i.e., a string of one or more 0 s) indicates temporal instability. The Stable Segment is defined as the longest consecutive sequence of adjacent Runs-plus-Gaps (e.g., {Run*_j_*, Gap*_j_*, Run*_j_*_+1_}), where each Run has a duration ≧ θ_Run_ and each Gap a duration ≤ θ_Gap_.

### E. Summary Statistics

For each file, BEATS computes nine Summary Statistics for the Stable Segment (referenced throughout the text as “A” through “I”).

**Figure 2:**
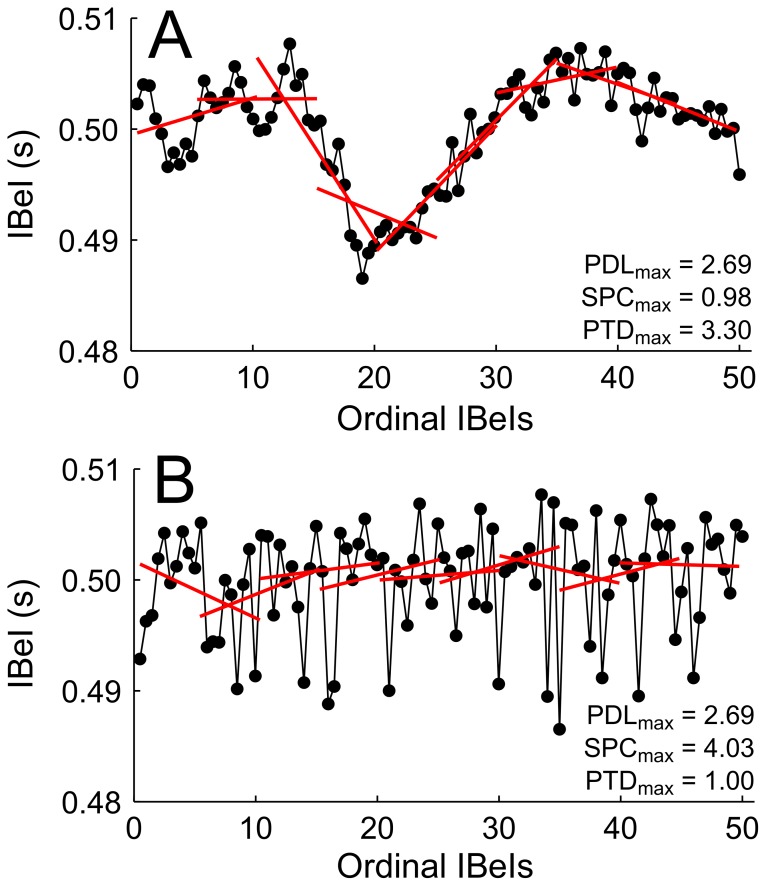
Illustrating the relationship between three measures of temporal instability. Two permutations of the same set of IBeIs are presented; both have identical central tendency and PDL_max_ statistics. The IBeI series in (A) exhibits temporal dependency, with gradual transitions from IBeI to IBeI. The IBeI series in (B) exhibits a more stochastic pattern of IBeI transitions. These differences in temporal structure are reflected in the SPC_max_ and PTD_max_ statistics.

- “Stable Duration”: the duration (in seconds) between the first and last timestamps of the Stable Segment.
- “Stable Percentage”: the Stable Duration as a percentage of the duration between the first and last timestamps of the IBeI series.
- “Run Percentage”: the percentage of the Stable Duration comprised of Runs. For example, if a Stable Segment was comprised of two Runs (each 30 s in duration) separated by a single Gap (2 s in duration), then the Run Percentage is 96.8%.
- “Estimated Tempo”: the central tendency (λ) of the entire IBeI series, converted to beats per minute (e.g., a λ of 1.0001 s yields an Estimated Tempo of 59.994 bpm).
- “Estimated Tempo Mismatch” (ETM): the signed percentage error of the tempo estimated by BEATS (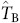, defined above) relative to the tempo estimate calculated by Echo Nest (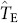; i.e., the tempo statistic queried from the MSD):
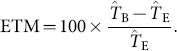
(5)
- “Estimated Meter”: a more precise operationalization of meter than the usual integer value (e.g., “4 beats-per-bar”). Specifically, for a Stable Segment with a bar timestamp series {*r_i_*, *r_i+1_*, *…*} and beat timestamp series {*b_j_*, *b_j+1_*, *…*}, let *n_i_* be the number of beat timestamps for which *r_i_ ≤ b_j_ <r_i+1_*. Estimated Meter is then taken as the mean of all *n_i_*. Only in the case when all *n_i_* have the same value will a true integer result (e.g., 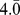), providing an easy way to identify audio files that have an unstable meter within the Stable Segment.
- “Maximum of Percentage Deviations from λ” (PDL_max_): The absolute value of the largest PDL (Eq. 1) across all Runs.
- “Maximum of Successive Percentage Changes” (SPC_max_): The absolute value of the largest SPC (Eq. 2) across all Runs. Although θ_Local_ sets the maximum tolerated amount of instability in PDL and SPC *a priori*, the largest *observed* PDL and SPC may in fact be smaller.
- “Maximum of Percentage Tempo Drift” (PTD_max_): the largest observed “short term drift” in tempo across all Runs, expressed as a percentage, and calculated as follows. First, within each Run, a series of 10-s windows is defined, with each successive window overlapping half of the previous window. Second, within each window, the best-fitting slope (i.e., linear tempo drift) through the IBeIs is found using least-squares linear regression Matlab’s polyfit (highlighted in red in the two example IBeI series shown in Figure 2). Third, for each calculated regression slope, the *y*-axis endpoints within window *w* are found, and expressed as percentage change (i.e., a “percentage of tempo drift”, PTD). In Figure 2A, for example, the best-fit slope in the 0 to 10 s window rises from *y* = .4997 to *y* = .5029 (yielding PTD = 0.65%), whereas the best-fit slope in the 10 to 20 s window falls from *y* = .5064 to *y* = .4897 (yielding PTD = −3.30%). Finally, PTD_max_ is taken as the largest absolute value of all PTDs across all Runs. For the IBeI series in Figure 2A, PTD_max_ = 3.30%.

Importantly, PDL_max_, SPC_max_, and PTD_max_ quantify partially independent aspects of temporal instability. The IBeI series in Figure 2B is in fact simply a random reshuffling of the IBeI series in Figure 2A, meaning that the two have identical means ( = 0.50), standard deviations ( = 0.005), and PDL_max_ ( = 2.69%) statistics. Their SPC_max_ and PTD_max_ statistics, however, are markedly different (by a factor of 4 and 3, respectively). Quantifying these three aspects of temporal instability provides a richer description of each IBeI sequence, as well as how IBeI sequences differ from one another.

### F. Implementation

To illustrate its various features, BEATS was run on the full Million Song Dataset using Initialization Thresholds of θ_Local_ = 5.0%, θ_Run_ = 10 s, and θ_Gap_ = 2.5 s. (The values of these thresholds, especially θ_Local_, should be considered *illustrative* rather than *prescriptive*; more will be said about this point in Section 1 of the Discussion.).

## Results

### 1. Individual Examples

**Figure 3:**
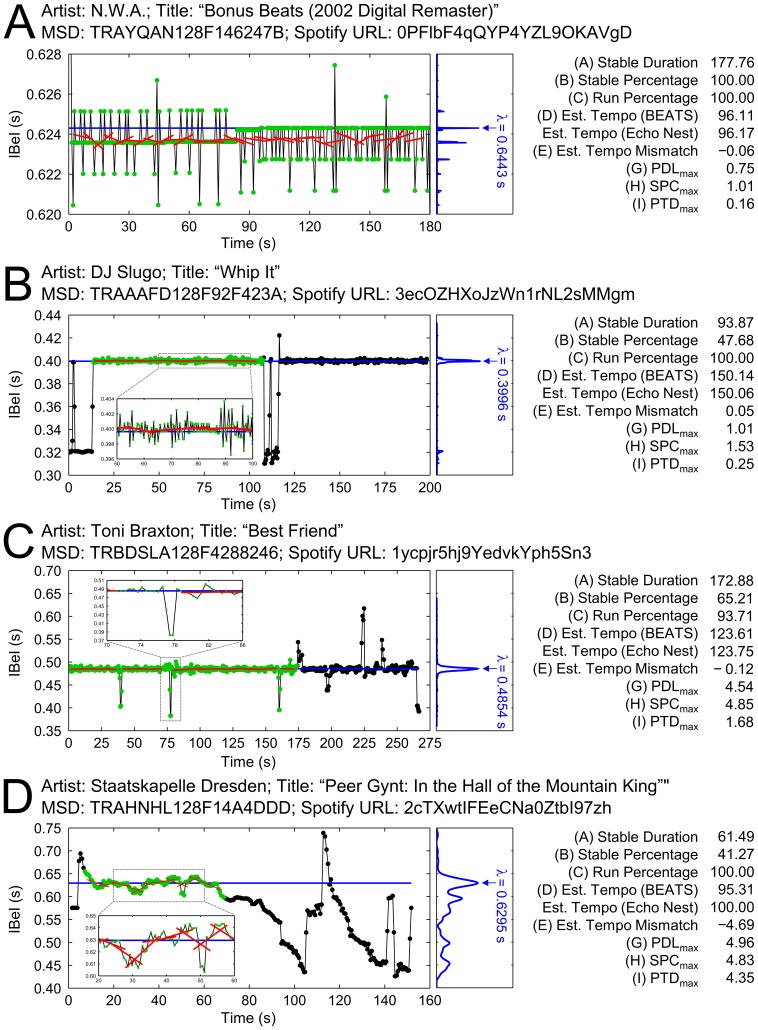
Four examples from the MSD illustrating the calculated Summary Statistics. IBeIs (*y*-axis) are plotted as a function of real time (*x*-axis). The central tendency (λ) of each IBeI distribution is obtained via adaptive KDE (right subpanel), plotted in blue. Slopes used to calculate PTD_max_ statistics are highlighted in red. The final Stable Segment (bridged across Gaps) is highlighted in green circles. Spotify URLs can be suffixed to https://play.spotify.com/track/ for listening.

**Figure 3** presents four individual MSD audio files that visually highlight one or more of the Summary Statistics. (All files had an Estimated Meter = 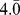.) Recordings of each audio file are available for listening via a Spotify URL.

In Figure 3A, the entire audio file consists of a repeating (looped) four-beat percussion riff. The IBeI series is highly regular, with nearly all successive IBeI differences being less than 2 ms. This audio file represents an “ideal” case: near-perfect isochrony from the first beat to the last, yielding very low values for the three Summary Statistics that quantify IBeI variability (PDL_max_, SPC_max_, and PTD_max_), as well as excellent agreement between BEATS’ Estimated Tempo and Echo Nest’s tempo estimate (a difference of less than one-tenth of 1%).

In Figure 3B, the audio file begins with a complex rhythm, to which a simple drum-and-cymbal rhythm (at approximately 150 bpm) at a higher frequency (pitch) and intensity (loudness) is added at the 13-s mark. This simple rhythm is removed at the 110-s mark, reintroduced at the 116-s mark, and remains in place until the end of the file at 199 s. It is this simple rhythm that drives the output of the Analyze beat detection algorithm. As such, the 94-s Stable Segment (identified by BEATS) is the longer of the two segments at that same tempo (the other being roughly 83 s). Within the Stable Segment, most IBeIs differ by only a few ms (similar to Figure 3A), yielding low values for the IBeI variability statistics. However, although the estimates of tempo by BEATS and Echo Nest again show excellent agreement, using the *entire* audio file in a motor synchronization paradigm (rather than just the Stable Segment) may prove challenging for some patients.

In Figure 3C, the Stable Segment is comprised of four distinct Runs bridged across three Gaps (at roughly 40 s, 77 s, and 160 s) that emerge as a consequence of unexpected syncopations in the voice (Gaps 1 and 2) or electric bass (Gap 3). PDL_max_ and SPC_max_ both have higher values than in the previous two examples, which might be expected as this audio file was recorded in a studio with session musicians (as opposed to synthesized on a computer, like the excerpts highlighted in Figures 2A and 2B) [92].

In Figure 3D, the *accelerando* for which the piece is famous is clearly visible in the IBeI plot; such an acoustic feature would, in theory, make for poor temporal stability. BEATS, however, was able to identify a 61-s Stable Segment where the tempo accelerated in less than 5% increments (as quantified by the “Maximum of Percentage Tempo Drift” statistic, PTD_max_).

Another feature of this IBeI series is notable. Although the *perceptual* tempo of the audio file continues to accelerate throughout its second half, the detected IBeI series (which had been tracking the quarter-note pulse) dramatically shifts from 0.42 s (at the 113-s mark) to 0.74 s (by the 116-s mark). Listening to the recording itself reveals a prominent change in timbre and intensity with the introduction of the chorus (and its strong accents on *alternating* quarter notes) at this point in the musical score (i.e., bar 49 in [93]). Although this musical event falls outside the Stable Segment, it raises an important point about the intimate dependency of BEATS on the beat tracking algorithm from which it takes its input data–a point detailed further in Section 1 of the Discussion.

### 2. Static Presentation of Summary Statistics

**Figure 4:**
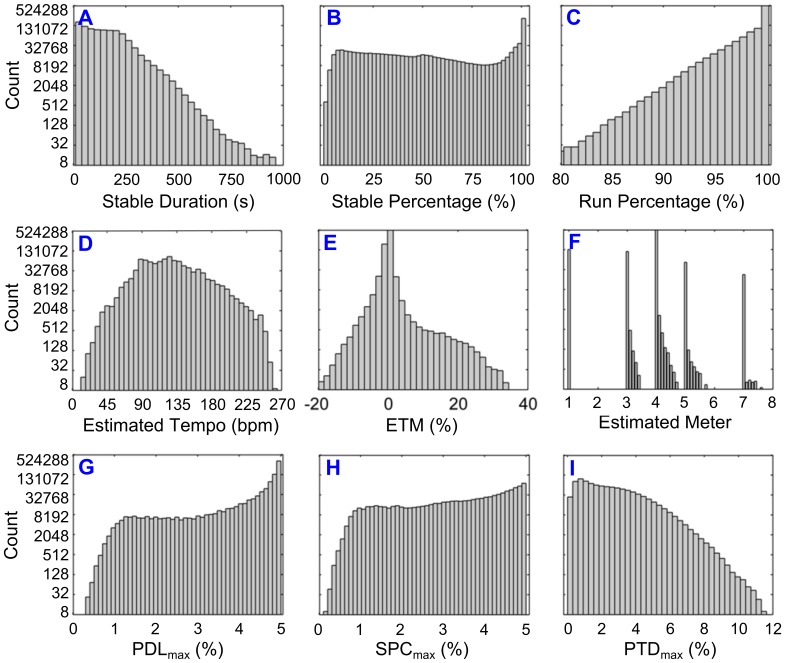
Histogram summaries of the nine Summary Statistics across the Million Song Dataset (*N* = 971,278), using log_2_ scaling along the *y*-axis to enhance visibility. Labels “A” through “I” correspond to the order in which Summary Statistics were defined in Section E of the Methods.

**Figure 4** presents a histogram (with log_2_ spacing along the *y*-axis for visual clarity) for each Outcome Statistic. The number of files actually summarized in Figure 4 is 971,278; the remaining files (i.e., 2.9% of the full MSD) did not have an identifiable Stable Segment which satisfied the Run Duration Threshold (i.e., were found to have less than 10 s of temporal stability).

An immediate question of interest concerns the agreement in “average” tempo as estimated by BEATS (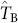) and Echo Nest (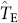). As revealed in **Figure 4E**, this match was generally quite high: 95% of all ETM percentage values fell within the interval [–2.20, 1.69]. That a vast majority of 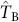 values differed from their 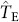 counterparts by less than the just-noticeable-difference for changes in tempo in isochronous IBeI sequences (cf. Section 1 of the Introduction) would seem, at first blush, to eliminate the need for BEATS entirely. Critically, however, agreement in terms of “average” tempo is only one piece of the puzzle, as it does address whether (and over what portion of the audio file) that tempo is *stable*–thus making that value statistically valid and experimentally useful.

In fact, Stable Percentage values (i.e., the percentage of each file’s duration that consisted of temporally stable of Runs that were separated by temporally unstable Gaps of no more than 2.5 s) varied widely across the MSD, as revealed in **Figure 4B**. Less than 22% of MSD files (*N* = 214,540) yielded a Stable Percentage = 100 (i.e., indicating temporal stability from the first detected beat to the last). This result has important consequences for “unsupervised” tempo-based playlist generation algorithms (e.g., [52]– [54]): only a fraction of audio files actually *maintain* their nominal tempo (i.e., the their Echo Nest tempo estimate) over their entire duration.

By contrast, if a user simply requires music that is temporally stable over a *minimum* duration (say, 90 s; useful for short gait training episodes or bouts of rhythmic exercise between rest periods) rather than its *entire* duration, a more optimistic picture emerges. As highlighted in **Figure 4A**, 61% of MSD files (*N* = 609,676) have a Stable Duration ≧90 s-nearly three times the number of MSD files that have a Stable Percentage = 100. Allowing BEATS to identify the Stable Segment within each audio file (rather than using the entire audio file *a priori*) yields a greater number of files that could be utilized in tempo-based playlists.

With respect to meter, agreement between BEATS and Echo Nest was very high, as highlighted in **Figure 4F**: for 99.6% (*N* = 967,226) files, the two estimates matched exactly (e.g., time_signature = 4 and Estimated Meter = 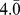). An unexpected result, however, also emerged: a substantial number of audio files (*N* = 21,412) yielded an Estimated Meter = 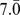. (This number was reduced to 11,164 when excluding audio files with a Stable Duration of less than 60 s.) This “odd” result was confirmed when comparing the time_signature statistic (i.e., Echo Nest’s own meter estimation) for these files; agreement was found in all cases. A cursory listening of these audio files revealed that the Estimated Meter value was, not surprisingly, inaccurate. Identifying misclassifications such as these will provide important “grist” to refine future beat tracking algorithms, a point further elaborated upon in Section 2 of the Discussion.

**Figure 5:**
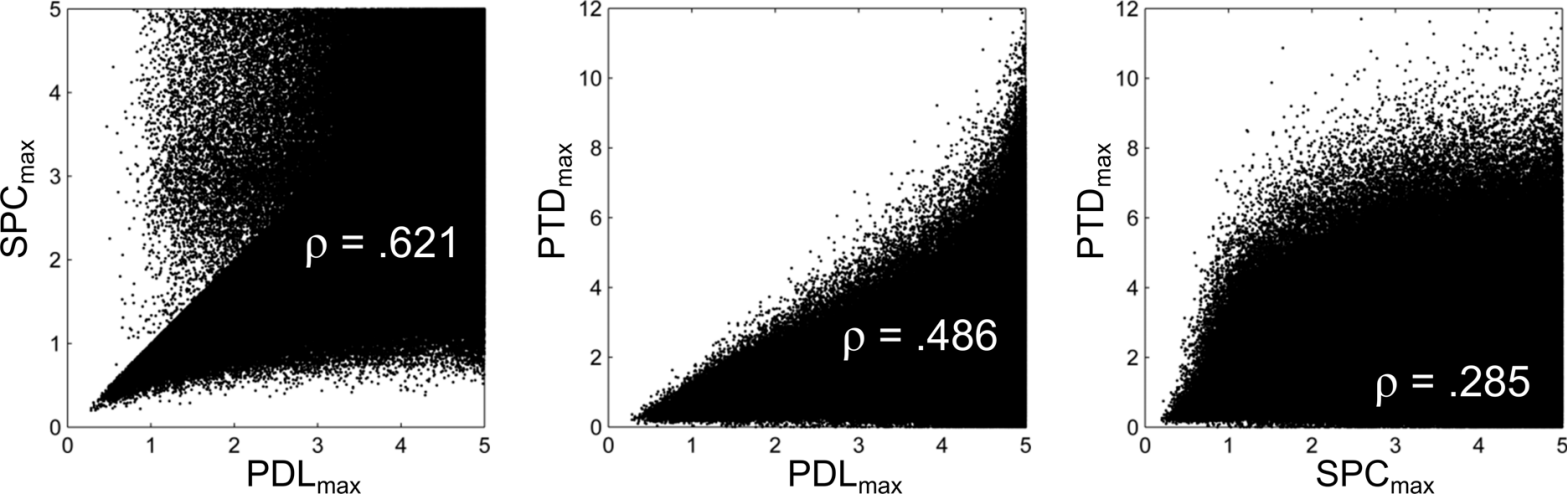
Pairwise scatter plot relationships (with associated Spearman correlation ρ values) for three BEATS Summary Statistics that quantify the stability of an IBeI series: PDL_max_, SPC_max_, and PTD_max_.

A final question pertains to correlations among the three Summary Statistics that most directly quantify the stability of an IBeI series: IBeI deviations from λ (PDL_max_), successive changes between IBeIs (SPC_max_), and IBeI drift within Runs (PTD_max_). **Figure 5** provides the answer, using scatter plots to visualize pairwise relationships between these three variables for the 609,676 MSD files with a Stable Duration ≧90 s. (This threshold was applied so that the scatter plot relationships would be less biased by Summary Statistics calculated from short excerpts of music.) Although the correlation between each pair of variables is positive (and “very” statistically significant given the large number of observations), it is clear that any one variable captures only a portion of what it means to be “temporally stable”.

### 3. Interactive Exploration of Summary Statistics

To more effectively interact with (and benefit from) the full set of Summary Statistics, an interactive tool is required. To this end, a LAMP-based (Linux, Apache, MySQL, PHP) web interface was developed. This interface, termed iBEATS (with a permanent URL at http://ibeats.smcnus.org/), integrates the full output of BEATS with three other valuable pieces of metadata: artist name, album release year, and descriptive genre tags.

For each item in the MSD, album release year was obtained by querying the 7digital application programming interface (API) (http://developer.7digital.com) using the MSD variable release_7digitalid. This yielded a total of 930,852 matches, a significant improvement upon the 515,576 files with a non-zero value in the MSD year variable [83].

For each unique artist in the MSD, a set of descriptive terms were pulled (MSD variable artist_terms) covering both high-level genre (e.g., “rock”, “electronic”, “heavy metal”) and specific subgenres (e.g., “garage rock”, “deep house”, “progressive metal”, etc.), as well as broad geographic descriptors (“brazilian”, “french”, “swedish”) and specific regional influences (e.g., “brazilian pop”, “french rap”, “swedish hip hop”), and up to 10 terms with an artist_terms_weight ≧0.5 for that particular artist were retained. The weight statistic, with values ranging from 0 to 1, reflects how descriptive a given term is with respect to the artist in question (as proprietarily determined by Echo Nest; cf. [94]), similar to a *term frequency-inverse document frequency* statistic. **Table 1** lists the 20 terms most frequently encountered artist terms in the MSD, tallying the number of artists and the number of songs associated with each term. (The Spearman correlation between these two item counts is ρ = .966 for the 1080 terms associated with at least 10 unique artists in the MSD.) The final number of MSD items which had valid tag data, year data, and a Stable Segment of at least 10 s was 902,081.

**Table 1:** The 20 most frequent artist_terms included in the Million Song Dataset.

Rank	Term	Number of artists	Number of songs
1	rock	13276	334709
2	electronic	10684	182981
3	pop rock	6455	185476
4	hip hop	6287	134748
5	electro	4921	88383
6	pop	4823	124291
7	indie rock	4699	102716
8	downtempo	4444	99307
9	disco	4241	104308
10	jazz	4192	117261
11	techno	4163	71281
12	alternative rock	4117	98359
13	tech house	3930	71697
14	trance	3504	53061
15	chill-out	3440	89033
16	folk rock	3283	91270
17	ballad	3228	111634
18	progressive house	3223	60806
19	deep house	3179	62626
20	blues	2905	84925

**Figure 6:**
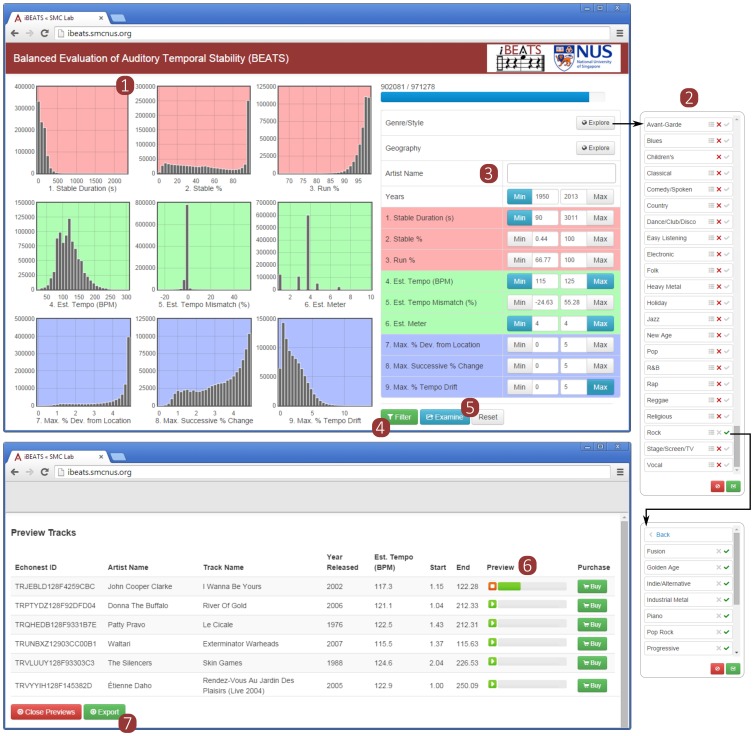
The iBEATS website (http://ibeats.smcnus.org/). The nine Summary Statistics are visualized using histograms (1). The user queries iBEATS by adjusting the numeric thresholds, browsing a two-level hierarchy of *Genre/Style* and *Geography* terms (2), and/or direct input to the *Artist Name* field (3). Filtering (4) reveals the number of candidate songs satisfying the query, which may then be further examined (5) and an audio sample previewed (6). The candidate playlist may then be exported (7) for subsequent use by a streaming music service (e.g., Spotify).

**Figure 6** presents a screenshot of an iBEATS query. The nine Summary Statistics are visualized using histograms, similar to Figure 2, and can be re-thresholded at liberty. To facilitate users’ ability to navigate musical space, 952 distinct artist terms were mapped onto one of two browsable, two-level hierarchies: one covering genre/style (with organization derived in part from www.allmusic.com/genres; e.g., “garage rock” is mapped to *Rock* → *Psychedelic/Garage*), and the other covering geography (roughly corresponding to continent and country; e.g., the term “suomi rock” is mapped to *Europe, Northern* → *Finland*). Additionally, specific artist names may be retrieved using text-based auto-completion (e.g., “ab” retrieves both *ABBA* and *Abbott & Costello* as options).

In the example shown in Figure 6, a playlist has been created for a hypothetical patient about to begin a gait rehabilitation paradigm. The following input parameters were used: all Rock genre songs from 1950 to the present, with a Stable Duration ≧90 s, Estimated Tempo between 115 and 125 bpm, Estimated Meter = 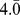, and PDL_max_, PSD_max_, and PTD_max_ all ≤5.0%. 19,725 audio files from the MSD satisfy this query, and are returned in a pop-up window; where available, 30-s audio previews are provided by making use of Echo Nest’s integration with 7digital audio previews [95]. (Note that the number of available files for a particular query is *scalable*: as BEATS expands further into the 35-million-item Echo Nest catalog of metadata, so too does the number of candidate songs satisfying that query.) The final, customized playlist (including, importantly, the starting and stopping time indices demarking the Stable Segment) may then be exported for subsequent handling by a streaming music player (e.g., Spotify; www.spotify.com), as described further in Section 2 of the Discussion.

## Discussion

Although many widely used beat tracking or tempo extraction algorithms, front-end software interfaces, and back-end metadata service providers offer point estimate statistics for the “average” tempo of an audio file, none has sought to systematically quantify the amount of *temporal instability* within an inter-beat interval (IBeI) series. Such an analysis is, we propose, acutely necessary to accurately design playlists for motor rehabilitation or rhythmic exercise paradigms, for which a stable beat is a prerequisite feature.

The proposed analysis tool, a “Balanced Evaluation of Auditory Temporal Stability” (BEATS), seeks to fill this need. The ultimate utility of BEATS, however, rests on (at least) two important caveats. The first caveat concerns the accuracy of the beat tracking algorithm; the second concerns the choice of thresholds used to define the Stable Segment.

### 1. Caveats

A first caveat, as noted in the Introduction, is that BEATS possesses no beat tracking capabilities itself; its raw material is a timestamp vector of beat and barline timestamps that had been previously detected by an external algorithm. For this reason, the idiosyncrasies of a particular beat tracking algorithm (or a systematic difference between two “competing” algorithms) will necessarily be reflected in whether and where BEATS identifies a Stable Segment of IBeIs. An algorithm’s beat tracking performance can be affected by both temporal (e.g., a complex rhythm loop) and non-temporal (e.g., recording quality) features of an audio file; examples of this were highlighted in Figure 3 and detailed in Section 1 of the Results.

Although this fact may make BEATS *conservative* (in that some audio files will be deemed to lack a Stable Segment of a “useful” minimum duration if many Gaps are present), such conservativeness may be beneficial in practice, as it will exclude pieces of music that may in fact be too challenging for listeners to synchronize with. (An ever-larger library of processed audio files will, of course, mitigate this conservativeness.) Indeed, the relationship between how a beat tracking algorithm performs and how listeners *themselves* perform when given a beat tracking task continues to drive developments in the field [79], [96]–[99]. The more closely an algorithm mimics human perception with respect to how it responds to temporal instability, the higher the quality of the Summary Statistics calculated by BEATS.

A second caveat is that the output of BEATS depends heavily on the choice of its Initialization Thresholds (cf. Section 3 of the Methods): the Local Stability Threshold (θ_Local_), Run Duration Threshold (θ_Run_), and Gap Duration Threshold (θ_Gap_). Of these three, θ_Local_ perhaps has the strongest influence over the likelihood of finding a Stable Segment with a “useable” duration (e.g., ≧90 s). In the present report, a value of θ_Local_ = 5.0% was selected. This value was chosen after a careful examination of the literature exploring just-noticeable differences (JNDs) within and between auditory temporal patterns (cf. Section 1 of the Introduction)–and determining that no prior reported threshold satisfied the constraints of the current project. Thus, the pattern of Summary Statistics obtained using θ_Local_ = 5.0% should be taken as illustrative rather than prescriptive. A conservative θ_Local_ value (e.g., 1.0%) would certainly *decrease* the number of available audio files with a useable Stable Duration, but at the same time *increase* the confidence that any audio files that “made the cut” were truly perceptually stable. Ultimately, adjusting both the Initialization Thresholds *and* the musical content (genre, artist, decade) to suit the needs and preferences of each target user (and the goals of the accompanying motor task) would seem the most prudent choice.

### 2. Future Directions

The primary aim of BEATS and iBEATS is to provide accurate statistics about tempo stability in a large collection of audio files, and to make that information easily accessible to users. Increasing the size of BEATS’ library (via access to Echo Nest metadata) to provide a greater collection of potential music stimuli is planned for the immediate future. Additionally, as noted by a reviewer, the manner in which genre/style terms are made available to a user *by* iBEATS may be as important as the statistics a user is hoping to obtain *from* iBEATS. Providing additional tools for musical “navigation” would offer enhanced accessibility and, in turn, widen the potential user base.

Although iBEATS itself is not viable as a means of delivering a rhythmic auditory cueing paradigm, we plan to author a mobile application that would (1) take a user’s input (artist, genre, tempo range, tempo stability thresholds, etc.); (2) query BEATS and obtain a candidate playlist; and (3) deliver that playlist using existing APIs authored by licensed streaming music services such as Deezer (http://developers.deezer.com/), Rdio (http://www.rdio.com/developers/), or Spotify (https://developer.spotify.com/). The ability to pair iBEATS with other mobile applications would offer novel ways to discover music; for example, by identifying a segment of audio using a music identification service (e.g., Shazam; http://www.shazam.com/) and then using BEATS to find music with similar temporal characteristics (a form of “query by example”; cf. [100]), or by utilizing a touchscreen-based “query by tapping” (cf. [101]) to more intuitively capture the desired movement rate.

In another vein, concurrent work from our laboratory [102] has sought to validate a mobile application to quantify the basic temporal dynamics of human gait in both healthy adults and Parkinson’s patients. A subject’s cadence (i.e., number of steps per minute) could then itself be used as an input parameter, creating a “query by walking” paradigm (which, although proposed previously [87], has yet to be explored within the music information retrieval literature).

### 3. Current Applications

Besides these future enhancements for “front end” users, current researchers may already benefit from BEATS. For researchers seeking to improve beat tracking algorithms, for example, BEATS could be used to identify audio files with “strange” IBeI patterns (e.g., Figure 3D) that may reflect an inherent limitation of a certain beat tracking algorithm, or to find those audio files with a sizable Estimated Tempo Mismatch (cf. Figure 4E).

BEATS could also prove useful with respect to identifying an algorithm’s misclassifications of meter (e.g., [103]) or tempo “octave” (e.g. [104]). Because the Stable Segment identified by BEATS within a given audio file possesses, by definition, a repeating acoustic pattern at some *rhythmic* level (e.g., eighth note), only a brief portion of the Stable Segment should be necessary for a human annotator to (1) indicate (i.e., tap) the *pulse* level (e.g., eighth note, quarter note, half note) they felt was most natural and (2) indicate whether the meter estimated by the algorithm (e.g., 3, 4) agreed with their own perceptions. This “accelerated” annotation process would greatly reduce the labor required to confirm these important statistics and identify misclassifications (e.g., the suspiciously high number of audio files with an “Estimated Meter = 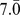
”, as noted in Section 2 of the Results). Such audio files would provide an immediate set of *diagnostic stimuli* that could be used to compare how beat tracking algorithms-particularly those informed by computational, psychological, and neurobiological models of how human listeners track patterns in time; for recent comprehensive reviews, see [12]–[14], [105], [106]–perform relative to listeners’ ground-truth tapping annotations. Fusing “bottom-up, data-driven” retrieval methods with “top-down, knowledge-based” models of human perception, cognition, and emotion remains a key focus for the field of music information retrieval (e.g., [43], [83]–[86]).

## Conclusion

We present a novel tool to quantify auditory temporal stability in recorded music (BEATS). An important departure that BEATS makes from other methods is that it seeks to identify the most temporally stable segment *within* an audio file’s inter-beat interval (IBeI) series, rather than derive a point estimate of tempo for the *entire* IBeI series. This increased flexibility enables BEATS to identify a greater number of candidate audio files for use in tempo-based music playlists. An online interface for this analysis tool, iBEATS (http://ibeats.smcnus.org/), offers straightforward visualizations, flexible parameter settings, and text-based query options for any combination of artist name, album release year, and descriptive genre/style terms. Together, BEATS and iBEATS aim to provide a wide user base (clinicians, therapists, caregivers, and exercise enthusiasts) with a new means to efficiently and effectively create highly personalized music playlists for clinical (e.g., gait rehabilitation) or recreational (e.g., rhythmic exercise) applications.
